# Pan-Cancer Analysis of the Cuproptosis-Related Gene DLD

**DOI:** 10.1155/2023/5533444

**Published:** 2023-11-30

**Authors:** Jiahui Lin, Guowei Wang, Sha Cheng, Yanan Hu, Huan Li, Wanjiang Feng, Xiaoming Liu, Canxia Xu

**Affiliations:** ^1^Department of Gastroenterology, The Third Xiangya Hospital of Central South University, Changsha 410013, China; ^2^Center for Experimental Medicine, Third Xiangya Hospital, Central South University, Changsha 410013, China

## Abstract

**Background:**

Cancer affects millions of people each year and imposes a huge economic and social burden worldwide. Cuproptosis is a recently discovered novel mode of cell death. The exact function of the cuproptosis-related gene dihydrolipoamide dehydrogenase (DLD) and its role in pan-cancer is unknown.

**Methods:**

Data were retrieved from the GTEx, TCGA, and multiple online websites. These data were used to assess the expression, prognosis, and diagnostic value of DLD in various tumors. The relationship of DLD with immune microenvironment immunomodulators, immune checkpoints, tumor mutational load (TMB), microsatellite instability (MSI), and oncology drug sensitivity was explored by correlation analysis.

**Results:**

The mRNA and protein expression of DLD differs in most cancers. Survival analysis showed that DLD was associated with prognosis with KIRC, KIRP, KICH, and UCS. DLD had a strong diagnostic value in KIRC, GBM, PAAD, and LGG (AUC > 0.9). DLD promoter methylation affects the aberrant expression of LIHC, LUSC, PAAD, READ, and THCA. DLD was negatively correlated with stromal score, immune score, and ESTIMATE score in UCEC, TGCT, LUSC, and SARC. In UCS, resting memory CD4 T cells and activated NK cells were significantly correlated with DLD expression. Significant correlations were also observed between DLD expression and immunomodulators, immune checkpoints, TMB, and MSI in various cancers. Importantly, we also identified a number of potential drugs that may target DLD.

**Conclusion:**

DLD expression is associated with a variety of tumor prognoses and plays an integral role in tumorigenesis, tumor metabolism, and immunity.

## 1. Introduction

Despite available surgical and chemotherapy treatments, cancer mortality remains high [1]. Cancer is driven by genetic changes and is a multifactorial, multistep, complex process [2, 3]. Meanwhile, metabolism is important in the carcinogenesis process, and in recent years, metabolism-targeted therapy has become an important part of tumor treatment [4, 5]. Tumorigenesis is complex; therefore, it is important to perform pan-cancer expression analysis of genes of interest and to assess their relevance to clinical prognosis and underlying molecular mechanisms.

DLD, also known as dihydrolipoamide dehydrogenase, is a component of the glycine cleavage system and an E3 component of the three alpha-keto acid dehydrogenase complexes [6, 7]. DLD is mainly localized in the mitochondria and, to a lesser extent, in the nucleus [8]. DLD variants are involved in multiple diseases [9, 10]. DLD deficiency is an autosomal recessive metabolic disorder [11, 12]. Clinically, affected individuals develop lactic acidosis and deterioration of neurological function due to the sensitivity of the central nervous system to defects in oxidative metabolism [13–15]. The DLD gene is a recently identified important gene associated with cuproptosis and regulates a specific metabolic pathway for cuproptosis [16].

Cuproptosis is a recently discovered programed cell death that triggers an uncommon method of cell death, which is essential for a variety of biological functions such as mitochondrial metabolism [17, 18]. Copper ions disrupt some specific mitochondrial metabolic enzymes that are more toxic in actively respiring cells. Copper-dependent death occurs through the direct binding of copper to the lipidated components of the tricarboxylic acid (TCA) cycle [19]. This leads to lipoylated protein aggregation and subsequent loss of iron–sulfur cluster proteins, resulting in proteotoxic stress and, ultimately, cell death [16]. The discovery of many cuproptosis-related genes may provide new perspectives on therapeutic approaches and prognoses for cancer patients. The DLD gene is a key gene that promotes copper death; however, the specific role of DLD in the prognosis and immune regulation of various cancers remains unexplored.

This study is the first to focus on the value of DLD in pan-cancer. Bioinformatic analysis was performed to assess the different DLD expressions in tissues and its possible association with cancer. DLD expression levels were significantly correlated with survival, immune cell infiltration, tumor mutational status, and microsatellite instability (MSI). DLD can be used as a new prognostic marker for various malignancies and as an indicator of cancer immunotherapy response.

## 2. Materials and Methods

### 2.1. Data Download and Analysis

Our analysis was based exclusively on data from existing databases. From the UNSC Xena website (https://xenabrowser.net/datapages/). Survival data were downloaded from the TCGA cancer dataset collected by Genomic Data Commons (GDC), while phenotypic information was obtained from the TCGA cancer dataset collected by GDC. The Human Protein Atlas (HPA) (http://www.proteinatlas.org/) database was obtained for immunofluorescence images of DLD expression in different tissues.

### 2.2. Differential Expression and Correlation Analysis of DLD

Differential expression studies using the Wilcox test to elucidate the general rules of transcriptome expression in pan-cancer. Box line plots and heat maps were used to illustrate differences in copper death-related gene expression between cancer and surrounding tissues.

### 2.3. Analysis of Tumor Microenvironmental Relevance of DLD

The ESTIMATE algorithm calculates microenvironment scores (using expression data to assess immune and stromal cells in tumor tissue). Different scores are applied to measure the microenvironment data: estimation score, stromal score, and immune score. The stromal score indicates the number of stromal cells (fibroblasts and vascular endothelial cells) in the tumor tissue; the immune score indicates the number of immune cells (T and B cells); and the estimated score indicates the sum of the stromal and immune scores. Higher estimate scores indicate lower purity of the tumor.

### 2.4. Drug Sensitivity Analysis

The Cell Miner database (https://discover.nci.nih.gov/cellminer/) provides matched mRNA sequencing and processed datasets of compound activity data (NCI-60 cell line set USA maintained by the National Cancer Institute) for drug sensitivity studies.

### 2.5. TIMER2 Database Analysis

TIMER2.0 (https://timer.cistrome.org/), based on the deconvolution approach, is an integrated web server that provides information on tumor-infiltrating immune cells from the gene expression profile of TCGA [20, 21]. TIMER 2.0 integrates six state-of-the-art algorithms, including xCell, TIMER, MCP-counter, EPIC, CIBERSORT, and quantTIseq, for immune infiltration estimation.

### 2.6. Genetic Variation Analysis

The cBioPortal tool (https://www.cbioportal.org/) was used to collect information on the frequency of alterations, mutation types, and mutation sites throughout the DLD protein structure.

### 2.7. Cell Culture and Quantitative Real-Time PCR (qRT-PCR)

GES-1 was obtained from Guangdong Hybribio Biotech Co., Ltd (China). AGS, MKN-45, and SGC-7901 were obtained from Hunan Fenghui Biotechnology Co., Ltd (China). All were incubated at 37°C in a humidified incubator with 5% CO_2_. Total RNA was isolated from cells using Trizol reagent (Invitrogen, USA) according to the manufacturer's protocol. cDNA was synthesized using HiScript II (Vazyme, China). Then, qRT-PCR of mRNA was performed on a real-time PCR system. GAPDH was used as a standard control for mRNA detection. Gene expression in PCR was obtained by log-transformation of CT values. DLD and GAPDH primers were synthesized by Tsingke Biotechnology Co., Ltd. (China). The primer sequences were as follows:

  GAPDH-forward: 5′-GGTCACCAGGGCTGCTTTA-3′;
  GAPDH-reverse: 5′-GGATCTCGCTCCTGGAAGATG-3′;
  DLD-forward: 5′-CTCATGGCCTACAGGGACTTT-3′;
  DLD-reverse: 5′-GCATGTTCCACCAAGTGTTTCAT-3′.

### 2.8. Statistical Analysis

Correlation between DLD expression and target was assessed using Spearman correlation tests, including tumor mutational load (TMB), MSI, and immune cell infiltration score. DLD expression levels were compared between tumor and normal tissues using *t*-test. All graphs were generated by the R package of ggplot2. Meanwhile, ^*∗*^,  ^*∗*^ ^*∗*^, and  ^*∗*^ ^*∗*^ ^*∗*^ indicate *p* < 0.05, *p* < 0.01, and *p* < 0.001, respectively.

## 3. Results

### 3.1. Differential and Coexpression Analysis of Cuproptosis-Related Genes

**Figure 1:**
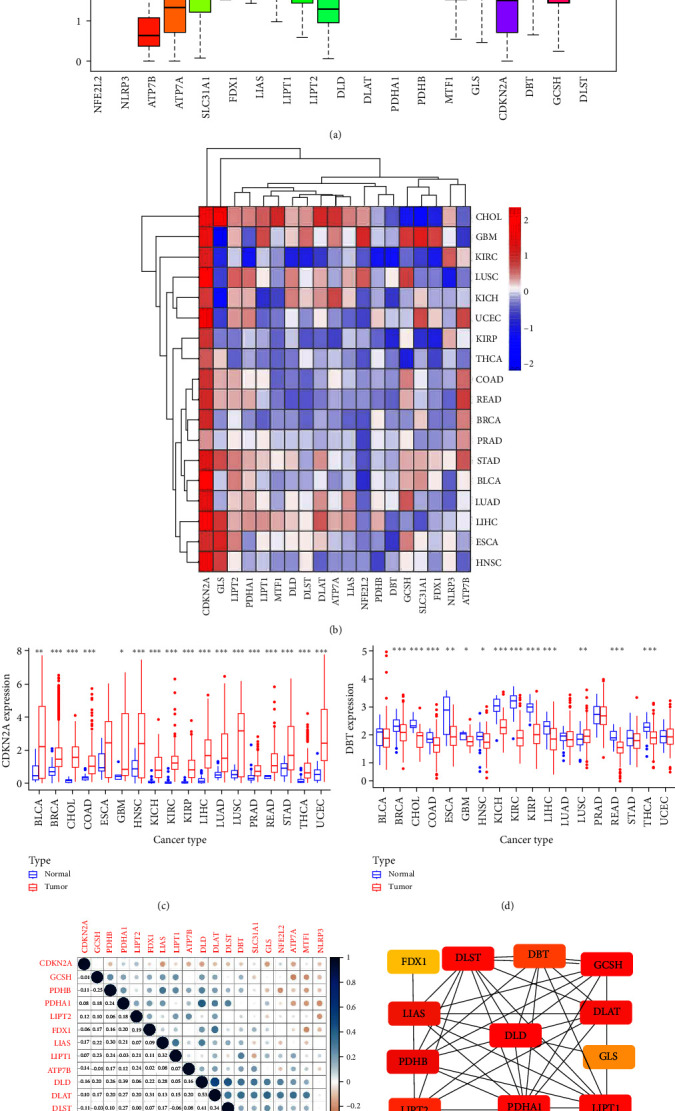
Differential expression and co-expression analysis of genes: (a) gene expression in pan-cancer; (b) heat map showing the expression levels of genes in 18 cancer types; the gradient of color represents log2 fold change (log FC) values; (c and d) differential expression of CDKN2A and DBT between tumors and adjacent normal tissues; (e) coexpression heat map demonstrating the coexpression relationship of cuproptosis-related genes; (f) PPI network constructed based on STRING database.

The expression of cuproptosis-related genes is shown in Figure 1(a). We found that DLST expression was high, while NLRP3 was at a low expression level across all tissues. The expression of genes varied in different tumors, and the expression of cuproptosis-related genes in different tumors is shown in the form of a heat map (Figure 1(b)).CDKN2A was significantly overexpressed in almost all tumors (Figure 1(c)), while DBT was significantly hypoexpressed in almost all tumors (Figure 1(d)). In addition, there was a general positive correlation between the coexpression of cuproptosis-related genes, indicating a general coexpression relationship of cuproptosis-related genes (Figure 1(e)). Notably, DLAT and DLD (correlation coefficient = 0.53), DLST and DLD (correlation coefficient = 0.41) were included. It has been found that DLD and DLST constitute the E3 and E2 components of the KGDH complex, which are closely linked to the mitochondrial ETC and regulate the cellular redox state [22]. Coexpression analysis of DLD revealed a potentially relevant network for DLD, which was consistent with external validation of the STRING database (Figure 1(f)). Based on this, the next section focuses on the role of DLD in pan-cancer.

### 3.2. Expression Levels of DLD in Various Normal and Cancerous Tissues

**Figure 2:**
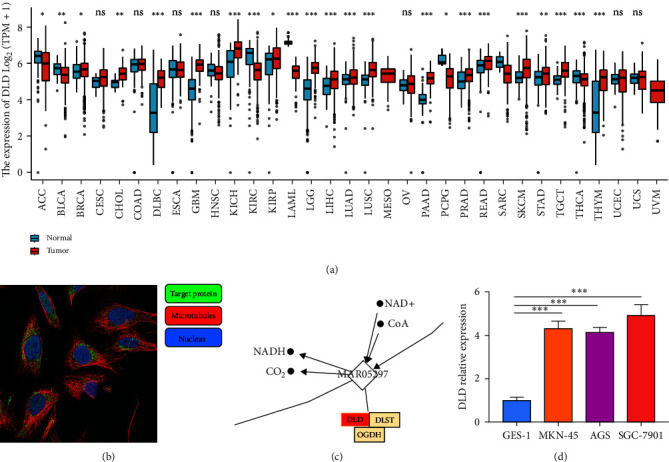
Expression levels of DLD in tumors of different tissues: (a) expression of DLD in different tumor types from TCGA and GTEx databases; (b) cellular immunofluorescence from the HPA database; (c) mining metabolic pathways for DLD effects in HPA-based databases; (d) mRNA expression of DLD in gastric cancer cell lines and gastric epithelial cells.

First, we investigated the expression of DLD in various normal and cancerous tissues. Using TCGA in combination with GTEx database data, we determined the mRNA expression levels of DLD. Significantly low expression in ACC, BLCA, KIRC, LAML, PCPG, THCA, and significant overexpression in BRCA, CHOL, DLBC, GBM, KICH, KIRP, LGG, LIHC, LUAD, LUSC, PAAD, PRAD, READ, SKCM, STAD, TGCT, THYM (Figure 2(a)). Immunofluorescence of cells from the HPA database revealed that DLD was mainly located in the mitochondria and partly in the nucleus (Figure 2(b)). From the HPA database, DLD was found to affect mainly the TCA cycle and glyoxylate/dicarboxylic acid metabolism (Figure 2(c)). We verified the expression of DLD in gastric cancer cell lines by cellular assays and found that the expression of DLD was significantly higher in gastric cancer cell lines (MKN-45, AGS, SGC7901) than in gastric epithelial cells (GES-1) (Figure 2(d)).

**Figure 3:**
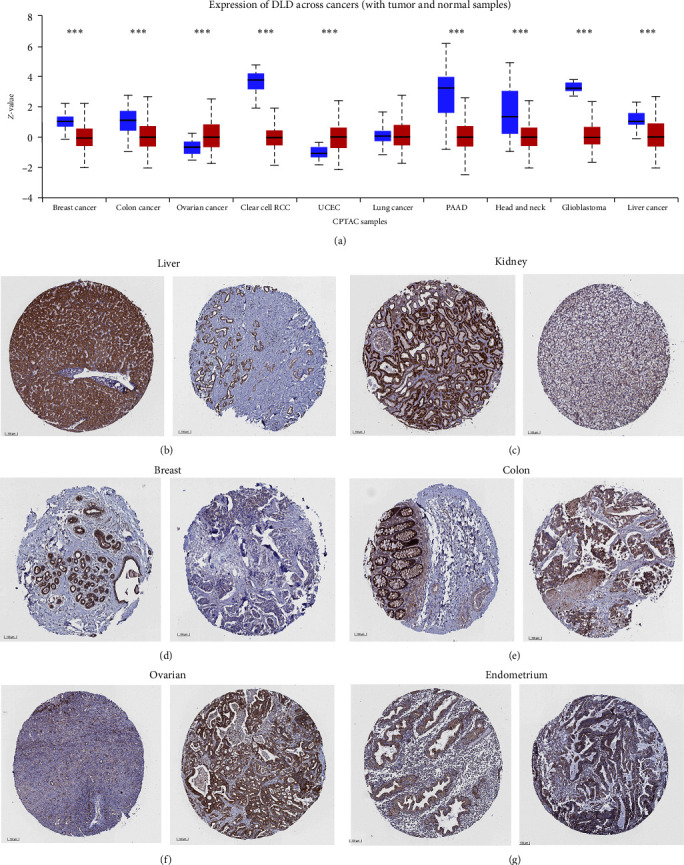
Protein expression profiles of DLD: (a) CPTAC dataset to analyze the protein expression of DLD; (b–g) representative immunohistochemical images of DLD proteins in normal and tumor tissues of the liver, kidney, breast, colon, ovarian, and endometrium from the HPA database.

In addition to transcription, we evaluated DLD at the protein level using large-scale proteomic data provided by the CPTAC dataset. We found that total protein expression of DLD was significantly lower in breast cancer, colon cancer, clear cell RCC, PAAD, head and neck squamous cancer, glioblastoma, and liver cancer compared to normal tissues. In contrast, DLD was significantly higher in UCEC and ovarian cancer compared to normal expression (Figure 3(a)). Representative immunohistochemical images from the HPA database showed that DLD was expressed lower in tumor tissues compared to normal tissues of the liver, kidney, chest, and colon (Figure 3(b)–3(e)). In contrast, the expression in normal ovarian and endometrial tissues was lower than that in tumor tissues (Figure 3(f) and 3(g)).

### 3.3. Analysis of Genetic Variants and Methylation Levels of DLD in Pan-Cancer

**Figure 4:**
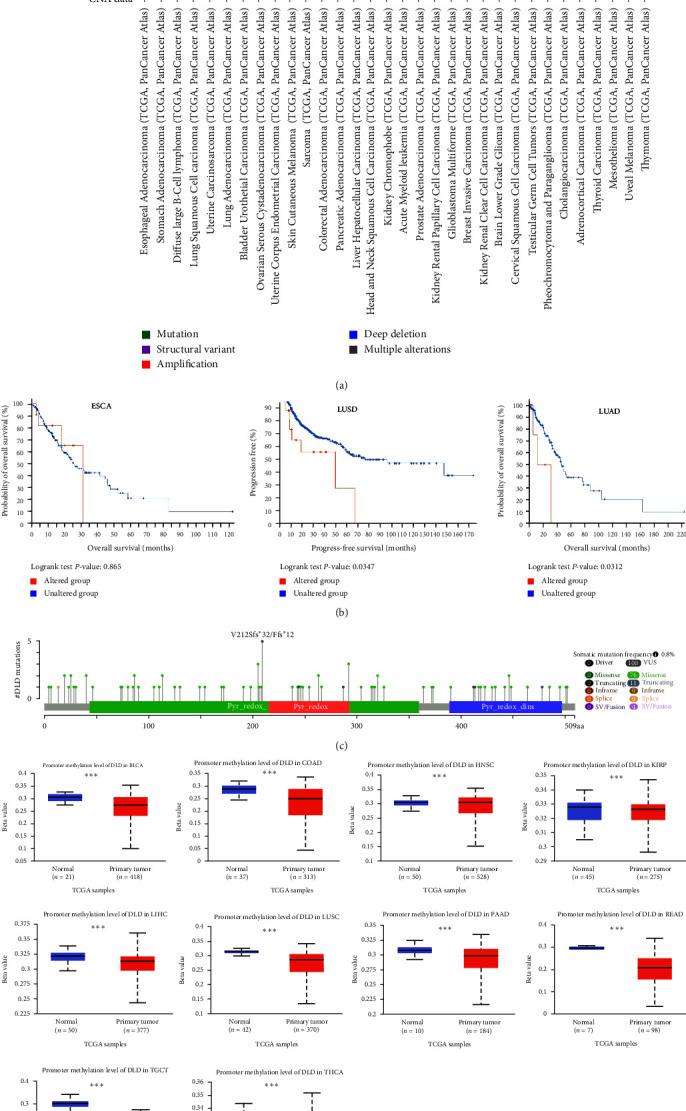
Genetic variation and DNA methylation of DLD in human cancers: (a) cBioPortal showing the frequency of alterations in DLD gene mutation types; (b) KM plots of DLD gene alterations in ESCA, LUSD, and LUAD; (c) mutation sites on DLD sequences; (d) methylation levels of DLD in cancer.

Human cancers develop due to the accumulation of genetic alterations. Therefore, we next explored DLD gene alterations in human tumor samples. Analysis of genomic analysis data from cBioPortal showed that the frequency of DLD gene alterations (6%) was highest in esophageal cancer, with the “amplified” type predominating at about 5.5% (Figure 4(a)), but the gene alterations did not affect the prognosis of patients with ESCA (Figure 4(b)). On the contrary, we found that genetic alterations in DLD significantly affected the prognosis in LUSD and LUAD, showing that the prognosis was worse in the genetically altered group (Figure 4(b)). We show the mutation sites on the DLD sequences, which include 100 VUS, 76 Missense, 11 truncating, 9 splices, and 4 SV/Fusion (Figure 4(c)). Alterations in DNA methylation patterns affect the expression profile of cancer-related genes. Therefore, we investigated the methylation levels of DLD in TCGA pancancer through the UALCAN database. The promoter methylation levels of DLD in BLCA, COAD, KIRP, LIHC, LUSC, PAAD, READ, and THCA were lower than those in the normal group (Figure 4(d)). In addition, the promoter methylation levels of DLD in HNSC and THCA were higher than normal, whereas no significant changes in DLD methylation levels were observed in other cancers. These results suggest that the aberrant expression of DLD in BLCA, COAD, KIRP, LIHC, LUSC, PAAD, READ, THCA, HNSC, and THCA may be attributed to changes in their promoter methylation.

### 3.4. Clinical Correlation Analysis of DLD

**Figure 5:**
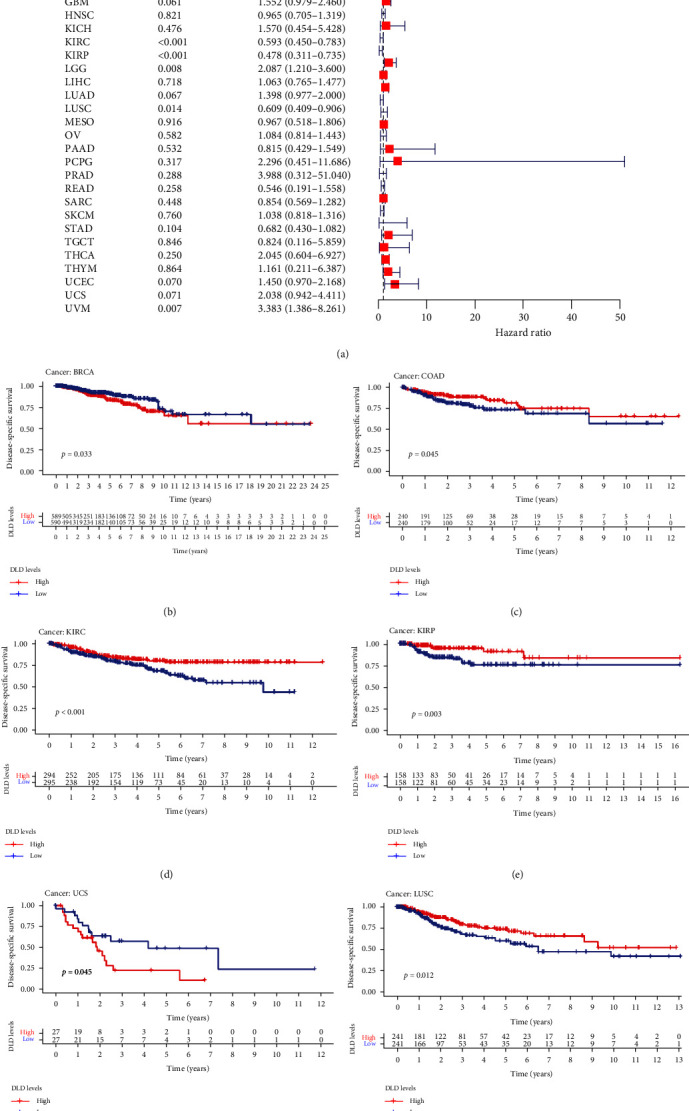
Relationship between DLD expression and DSS in cancer patients: (a) forest plot of DLD risk ratio in pan-cancer; (b–g) Kaplan–Meier survival curves of DSS in patients with BRCA, COAD, KIRC, KIRP, UCS, and LUSC.

We assessed the correlation between the expression levels of DLD and its clinical diagnostic and prognostic value in various cancers. First, we analyzed the overall survival of the disease. Univariate COX regression and K–M analysis showed that DLD had prognostic value in KIRC, KIRP, KICH, and UCS. DLD is associated with poor prognosis in UCS and KICH and is a tumor suppressor of KIRC and KIRP (see Figure S1). However, OS may be influenced by noncancer-related deaths during follow-up. Therefore, data on the correlation between disease-related survival (DSS), progression-free interval (PFI), and DLD expression were analyzed in various cancers. DSS analysis showed that high expression of DSS predicted poor prognosis in BLCA and UCS, while in COAD, KIRC, KIRP, and LISC were protective factors (Figure 5). PFI analysis suggested that upregulated DLD expression was a better prognostic value for KIRC and KIRP (see Figure S2). Combining prognostic analysis, we speculate that DLD can affect the prognosis of multiple tumors, especially in KIRC. Meanwhile, we explored the diagnostic value of DLD in pan-cancer and found a strong diagnostic value (AUC > 0.9) in KIRC, GBM, PAAD, and LGG (see Figure S3).

### 3.5. DLD Is Associated with Tumor Mutational Load, Microsatellite Instability, and Immune Checkpoints

**Figure 6:**
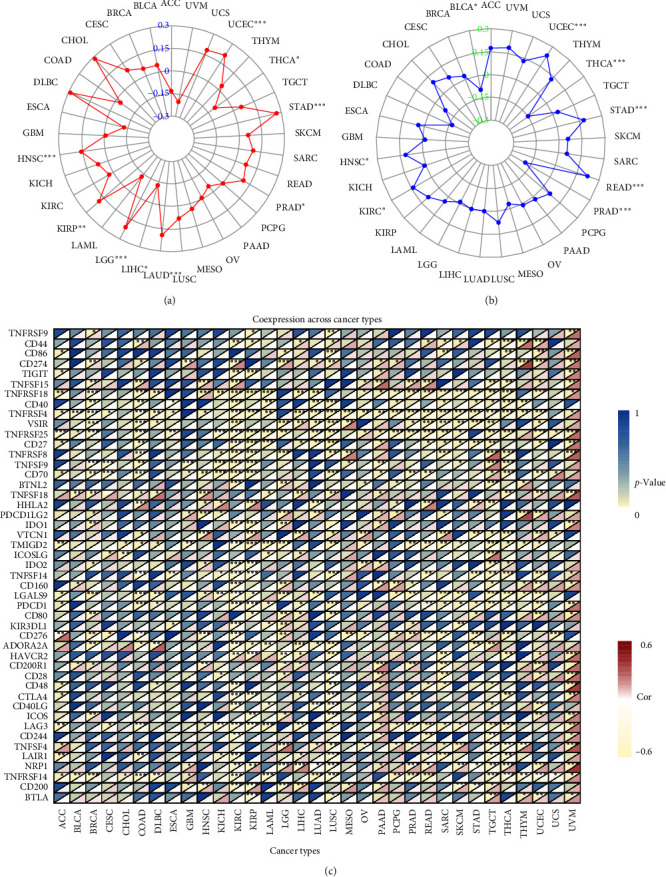
Relationship between TMB, MSI, immune checkpoint, and DLD expression in different tumors: (a) correlation between TMB and DLD; (b) correlation between MSI and DLD; (c) heat map of the relationship between DLD and known immune checkpoints.

TMB and MSI are potent prognostic biomarkers in a variety of tumors and can independently predict the efficacy of tumor immunotherapy. The correlation between DLD expression and TMB was significant (p < 0.05). DLD expression was positively correlated with TMB in UCEC, STAD, PRAD, LUAD, LGG, KIRP, and HNSC, while negatively correlated in THCA and LIHC (Figure 6(a)). Correlation analysis of DLD expression with MSI showed that DLD was significantly positively correlated with MSI in UCEC, STAD, READ, KIRC, and HNSC, while negatively correlated in THCA, PRAD, and BLCA (Figure 6(b)). We further explored the association between DLD expression and pan-cancer immune checkpoints. Interestingly, we found that DLD was positively correlated with PD1 (PDCD1) and cytotoxic T lymphocyte antigen 4 (CTLA-4) in PAAD, THYM, and UVM (Figure 6(c)). DLD was closely associated with PD-L1 (CD274) in BRCA, COAD, GBM, KIRC, LGG, LUAD, LUSC, PAAD, PRAD, PCPG, THYM, UCEC, and UVM. These results suggest that DLD may regulate different immune responses in different cancer types.

### 3.6. Relationship between DLD and Immune Cell Infiltration

**Figure 7:**
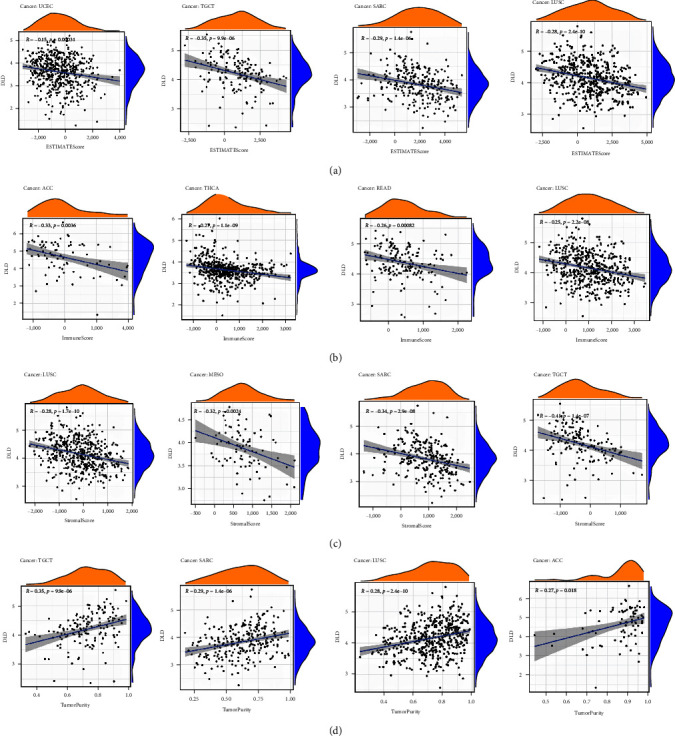
Correlation between DLD and StromalScore, ImmuneScore, ESTIMATEScore, and TumorPurity: (a) ESTIMATEScore; (b) ImmuneScore; (c) StromalScore; (d) TumorPurity.

The immune cells contained in the tumor microenvironment have the effects of promoting cancer and resisting tumor, which can affect the progress and recurrence of tumor [23]. The relationship between immune-related scores and DLD expression was analyzed. The correlation between StromalScore, ImmuneScore, ESTIMATEScore, and TumorPurity of the top four tumors is shown in Figure 7. It is obvious that DLD was negatively correlated with StromalScore, ImmuneScore, and ESTIMATEScore of UCEC, TGCT, LUSC, SARC, etc. In addition, we explored the correlation between immune cell infiltration and DLD using the CIBERSORT immune cell infiltration algorithm. Immune cells significantly correlated with DLD expression in different tumors (p < 0.01, | *R* | > 0.3) can be found as Figure S4. In KICH, T cells CD4 memory resting were negatively correlated with DLD (*R* = 0.65, *p* = 2.4e–05). Mast cells resting (*R* = −0.43, *p*=0.0094) and NK cells activated (*R* = − 0.4, *p*=0.015) were significantly correlated with DLD in UCS. Timer database to further analyze the B cell, T cell, and macrophage infiltrations of DLD in different tumors (see Figure S5). DLD was negatively correlated with CD4+ Th1 cells and positively correlated with CD4+ Th2 cells in most of the tumors.

### 3.7. Drug-Sensitivity Analysis in Pan-Cancer

**Figure 8:**
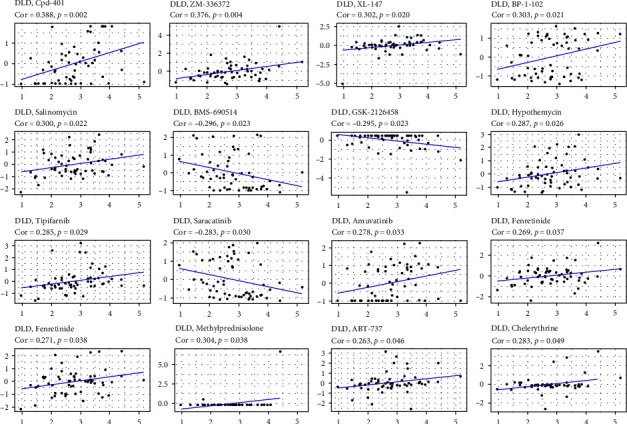
Association of DLD expression with drug sensitivity in different cancers.

The degree of gene expression can change the sensitivity of tumor cells to some drugs. Correlation analysis of drug sensitivity of various cancer cell lines by using the Cell Miner database. DLD was positively correlated with drug sensitivity to Cpd-401 and Salinomycin, implying that patients with high DLD expression may be more likely to receive antitumor therapy with Cpd-401 and Salinomycin. In contrast, DLD was negatively correlated with sensitivity to BMS-690514 and Sarcatinib, indicating an increased risk of drug resistance to BMS-690514 and Sarcatinib with increasing levels of DLD expression (Figure 8).

## 4. Discussion

Targeting copper metabolic homeostasis and inducing copper death holds great promise in the field of tumor therapy [24]. Pan-cancer analysis attempts to compare genomic and cellular changes observed in different tumor forms in order to infer common mechanisms shared by different cancer species. In this study, we discuss the correlation between DLD expression and TCGA tumor characteristics, including clinical significance, drug sensitivity, DNA methylation, genetic alterations, and immune landscape.

We based on the correlation analysis of copper death-related genes NFE2L2, DLST, SLC31A1, DLD, DLAT, PDHA1, PDHB, DLD, GLS, ATP7B, ATP7A, LIAS, LIPT1, LIPT2, MTF1, CDKN2A, DBT, GCSH. STRING database analysis in the potential correlation network of DLD was constructed. This prompted us to further explore the role of DLD in pan-cancer. Previous studies have shown that DLD is associated with affecting multiple tumors. DLD plays a key role in melanoma progression and proliferation, promoting melanoma growth and tumor proliferation in vivo [25]. DLD is involved in iron death in HNSC [26]. Autoantibodies of DLD can be used as a novel diagnostic marker for ovarian cancer [27]. Currently, DLD has not been extensively studied in the cancer field.

We compared the expression of DLD at mRNA and protein levels through several databases. DLD is aberrantly expressed in multiple tumors. High expression of DLD in gastric cancer cells was identified by cellular experiments. The HPA database reveals that DLD is mainly located in mitochondria and affects metabolic pathways. However, the role of DLD in cancer cell metabolism has not been extensively studied yet. It has been shown that DLD downregulation significantly increases *α*-ketoglutarate and decreases succinate, and DLD inhibition can decrease TCA cycle downstream metabolites, leading to altered mitochondrial energy metabolism in melanoma [25].

Gene mutations and epigenetic modifications can induce aberrant gene expression during tumorigenesis. In this study, DLD gene alterations occurred in most cancer types, mainly by amplification. We found that genetic alterations in DLD significantly affected the prognosis in LUSD and LUAD, and the results showed that the prognosis was worse in the genetic alteration group. We speculate that DLD gene alterations play an important role in the development of lung cancer and thus affect patient prognosis. Aberrant DNA methylation is a common epigenetic feature of cancer. We then investigated the methylation levels of DLD in pan-cancer. The mRNA expression levels of DLD were significantly higher in LIHC, LUSC, PAAD, and READ than in normal samples, while lower promoter methylation levels were observed. Meanwhile, mRNA expression levels of DLD were significantly lower in THCA, and their promoter methylation levels were higher. Hypermethylation usually silences or inactivates tumor suppressor genes in cancer [28]. Therefore, we suspect that the aberrant expression of DLD in LIHC, LUSC, PAAD, READ, and THCA may be due to their promoter methylation.

DLD was found to affect the prognosis of multiple tumors, especially in KIRC, by analyzing OS, DSS, and PFI in various tumors. Meanwhile, we explored the diagnostic value of DLD in pan-cancer and found a strong diagnostic value in KIRC, GBM, PAAD, and LGG. It indicates that DLD is a potential diagnostic and prognostic marker for KIRC, and high expression of DLD predicts a better prognosis.

Understanding the composition of immune cells in tumor tissues will help to find new approaches to cancer treatment and improve the efficiency of ICB therapy. We analyzed the relationship between immune-related scores and DLD expression. The expression level of DLD was negatively correlated with StromalScore, ImmuneScore, and ESTIMATEScore of UCEC, TGCT, LUSC, SARC, etc. In addition, CD4 T cells memory resting and activated NK cells were significantly correlated with DLD in UCS. Whether resting memory CD4 T cells and activated NK cells play an important role in the poor prognosis of UCS predicted by high DLD expression needs to be further investigated.

TMB can be used as a prognostic and predictive biomarker of immunotherapeutic response in human cancer [29]. MSI is also a key biological marker of the immune checkpoint inhibitor (ICI) response. The US Food and Drug Administration has approved MSI-high status as predictive biomarkers to guide the clinical use of ICIs in certain cancers [30]. DLD expression is positively correlated with TMB and MSI in most tumors. Immune checkpoints, such as CTLA-4 and programed cell death 1, are surface proteins that are expressed primarily on T cells. When interacting with ligands expressed on antigen-presenting cells, they inhibit the initiation, duration, and magnitude of the immune response [31]. Cu ion carrier disulfiram can induce stabilization of PD-L1 by overloading cancer cells with Cu [32]. DLD is closely associated with PD-L1 in BRCA, COAD, GBM, KIRC, LGG, LUAD, LUSC, PAAD, PRAD, PCPG, THYM, UCEC, and UVM. DLD regulates different immune responses in different cancer types, which is useful for guiding the clinical application of immune checkpoint inhibitors in certain cancers.

In addition, we explored the role of DLD as a potential target for tumor therapy by analyzing the correlation between DLD expression and the drug sensitivity to reveal potential therapeutic agents. Among them, DLD was positively correlated with the drug sensitivity of Cpd-401 and Salinomycin, implying that patients with high DLD expression may be more likely to receive antitumor therapy with Cpd-401 and Salinomycin. In contrast, DLD was negatively correlated with sensitivity to BMS-690514 and Sarcatinib, indicating an increased risk of drug resistance to BMS-690514 and Sarcatinib with increasing levels of DLD expression. This implies that dysregulation of DLD may lead to antineoplastic drug resistance.

The present study shows the results of comprehensive pan-cancer analysis data of DLD. The expression, prognosis, diagnosis, immune-related analysis, immune checkpoints, TMB, and MSI of DLD were combined for analysis. This information contributes to the understanding of the function of DLD in cancer development and its role in immunology. However, more experimental studies are needed to explore the specific mechanisms underlying the role of DLD in cancer.

## Data Availability

Expression matrix data were obtained from UCSC Xena (https://xenabrowser.net/datapages/), which are publicly available. Also, analyzed in conjunction with the following public online databases (http://www.proteinatlas.org/;https://discover.nci.nih.gov/cellminer/;https://timer.cistrome.org/;https://www.cbioportal.org/). More information can be accessed from correspondence authors.

## Supplementary Materials

Supplementary Materials Figure S1: relationship between DLD expression and OS in cancer patients. Figure S2: relationship between DLD expression and PFI in cancer patients. Figure S3: diagnostic value of DLD in various tumors. Figure S4: correlation of DLD with immune cell infiltration. Figure S5: the B cell, T cell, and macrophage infiltrations of DLD in different tumors.
