# Supplementary material for: Pan-Cancer Analysis of the Cuproptosis-Related Gene DLD
Source: Mediators Inflamm. 2023 Nov 30;2023:5533444. doi: 10.1155/2023/5533444 (PMC10703539; doi:10.1155/2023/5533444)
Supplement: Supplementary Materials — Figure S1: relationship between DLD expression and OS in cancer patients. Figure S2: relationship between DLD expression and PFI in cancer patients. Figure S3: diagnostic value of DLD in various tumors. Figure S4: correlation of DLD with immune cell infiltration. Figure S5: the B cell, T cell, and macrophage infiltrations of DLD in different tumors. [file 5533444.f1.pdf]

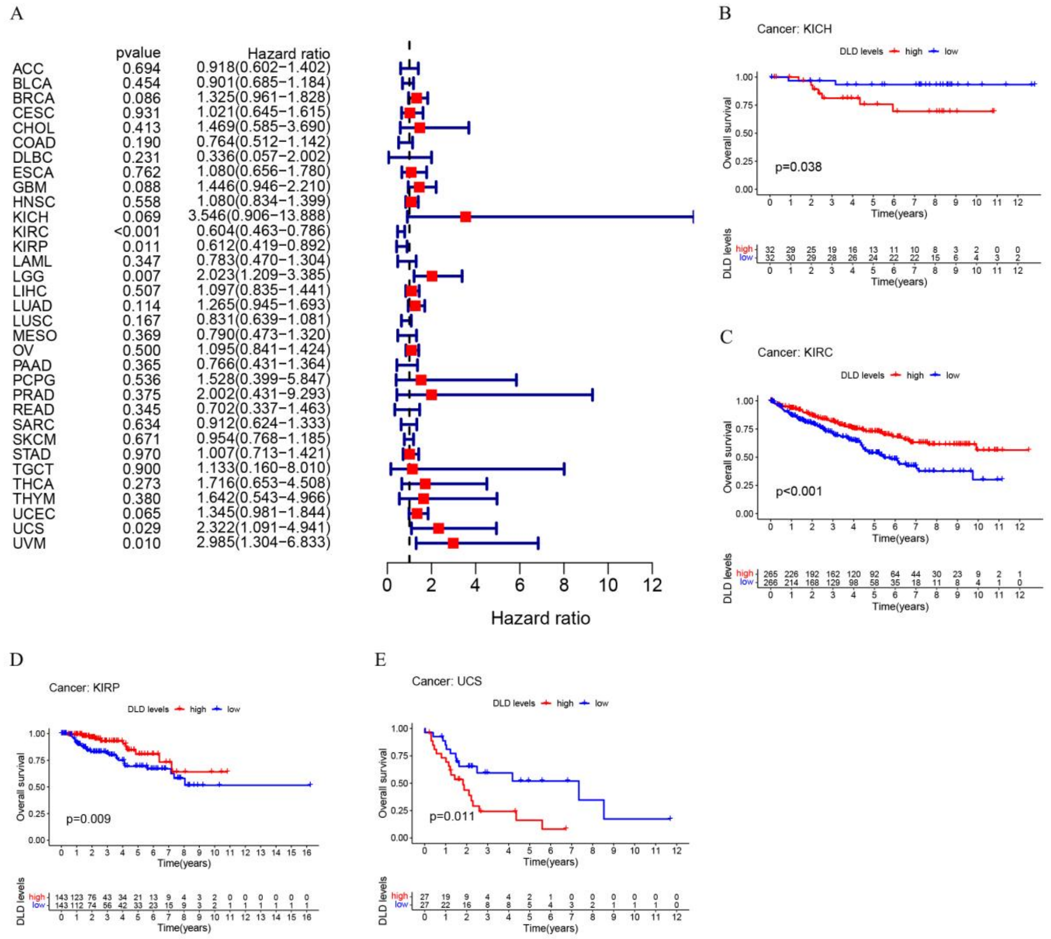

Supplementary Figure 1 | Relationship between DLD expression and OS in cancer patients. (A)Forest plot of DLD risk ratio in pan-cancer; (B-E) Kaplan-Meier survival curve of OS in KICH , KIRC,KIRP and UCS patients.

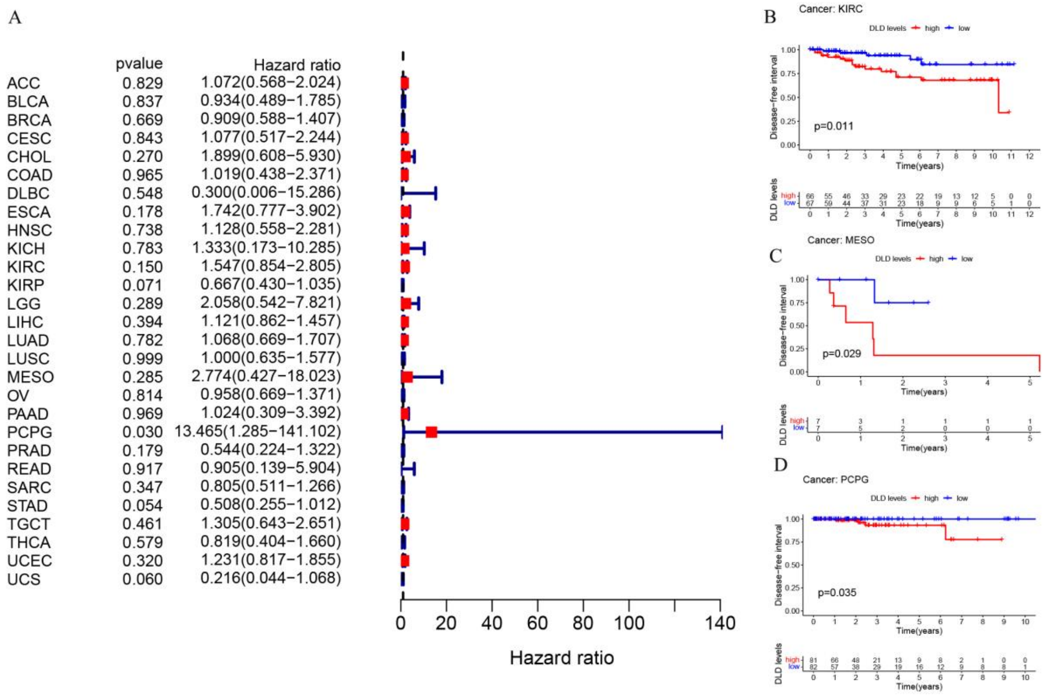

Supplementary Figure 2 | Relationship between DLD expression and PFI in cancer patients. (A) Forest plot of DLD risk ratio in pan-cancer; (B-D) Kaplan-Meier survival curve of PFI in KIRC ,MESO and PCPG patients.

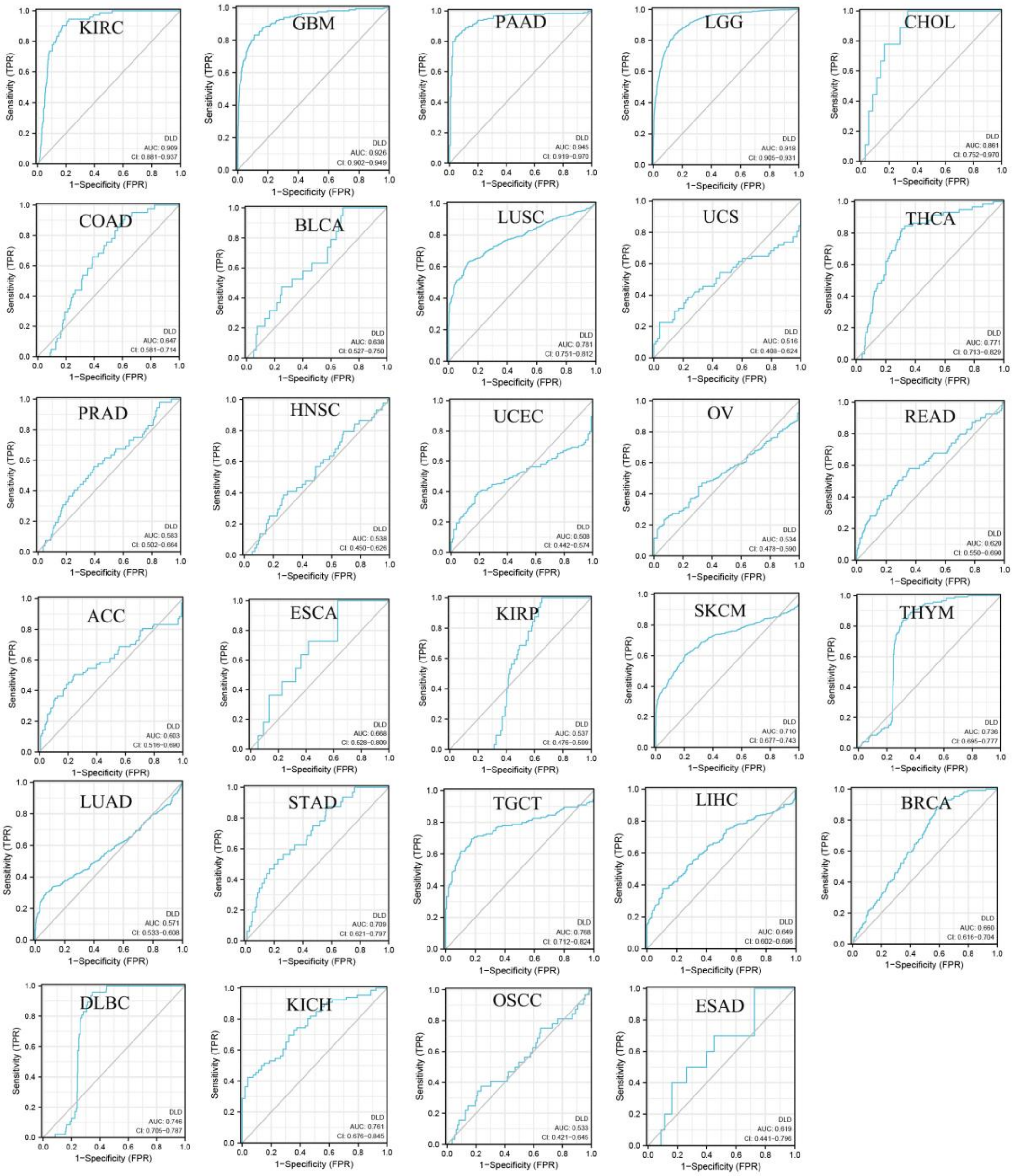

Supplementary Figure 3| Diagnostic value of DLD in various tumors

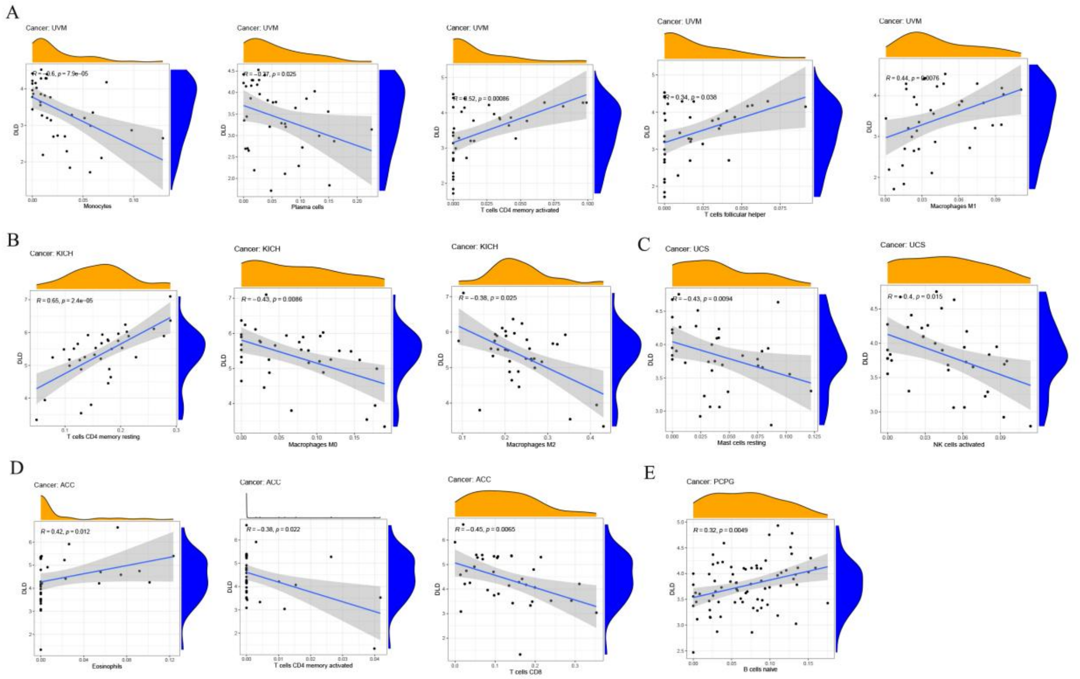

Supplementary Figure 4 | Correlation of DLD with immune cell infiltration. (A-E) Correlation analysis of DLD with immune cell infiltration in UVM, KICH, UCS, ACC and PCPG.

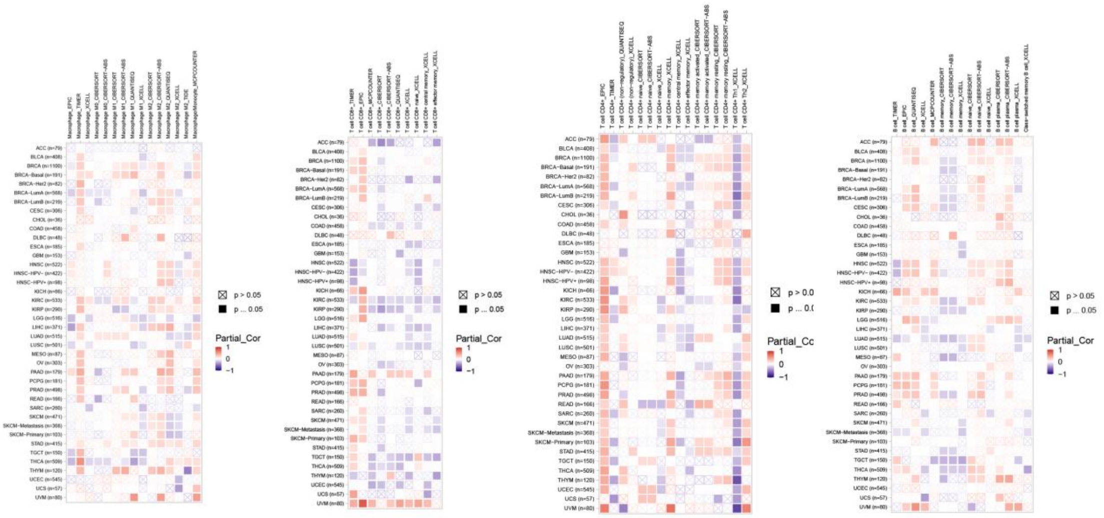

Supplementary Figure 5| The B cell, T cell, and macrophage infiltrations of DLD in different tumors
